# Association of hypertension and hypertriglyceridemia on incident hyperuricemia: an 8-year prospective cohort study

**DOI:** 10.1186/s12967-020-02590-8

**Published:** 2020-10-31

**Authors:** Yuan Zhang, Miaomiao Zhang, Xiawen Yu, Fengjiang Wei, Chen Chen, Kai Zhang, Shuzhi Feng, Yaogang Wang, Wei-Dong Li

**Affiliations:** 1 School of Public Health, Tianjin Medical University, Tianjin, 300070 China; 2 Department of Genetics, School of Basic Medical Sciences, Tianjin Medical University, Tianjin, 300070 China; 3 Tianjin General Hospital, Tianjin Medical University, Tianjin, 300052 China

**Keywords:** Hypertension, Hypertriglyceridemia, Hyperuricemia, Prospective cohort study

## Abstract

**Background:**

Hypertension and high triglyceride are two of the most important risk factors for hyperuricemia. Epidemiological records show that hypertension and dyslipidemia often coexist and may significantly increase the risk of target organ damage. However, their combined effect on incident hyperuricemia is poorly understood. Thus, we aimed to investigate the separate and combined effect of hypertension and hypertriglyceridemia on the incidence of hyperuricemia.

**Methods:**

A prospective cohort study of 6424 hyperuricemia-free participants aged 20 to 94 years between August 2009 and October 2017 was performed at Tianjin General Hospital of China. Participants were categorized into four groups by combining hypertension and hypertriglyceridemia status at baseline. The restricted cubic spline fitting Cox regression model was used to evaluate the relationship between blood pressure and triglyceride and hyperuricemia. Cox regression models were performed to calculate hazard ratios (HRs) and 95% confident intervals (CIs) to estimate baseline factors and their association with the incidence of hyperuricemia. A Kaplan–Meier survival analysis was performed to compare the incidence of hyperuricemia among subjects in each separate and combined hypertension and hypertriglyceridemia group.

**Results:**

During the 8-year follow-up period, 1259 subjects developed hyperuricemia (20.6%). There existed positive relationships between blood pressure and triglyceride levels and hyperuricemia. This risk factor arising from a combination of the two (HR, 3.02; 95% CI 2.60–3.50) is greater than that from hypertension (HR, 1.48; 95% CI 1.28–1.71) or hypertriglyceridemia (HR, 1.84; 95% CI 1.55–2.18) separately. The Kaplan–Meier survival analysis indicated that combined effect of hypertension and hypertriglyceridemia may predict higher onset of hyperuricemia.

**Conclusion:**

The combined effect of hypertension and hypertriglyceridemia on the risk of hyperuricemia is much stronger than that by hypertension or hypertriglyceridemia separately. Hypertension combined with hypertriglyceridemia may be an independent and powerful predictor for hyperuricemia.

## Background

Hyperuricemia (HUA), a major risk factor for gout, affects 13.3% of the Chinese population [1], 21.4% of the U.S [2]. and 25.8% of the Japanese [3]. Asymptomatic hyperuricemia has also been identified as an independent risk factor for the development and progression of cardiovascular disease, coronary heart disease mortality and all-cause mortality [4–6]. So far, however, a clearly pathological mechanism of hyperuricemia has not been fully elucidated, and long-term anti-hyperuricemic therapy carries both costs and risks. Thus, identifying people at high risk of hyperuricemia and providing early prevention is quite important.

To promote prevention of hyperuricemia, risk factors in the chain of causation must be identified. Some previous studies have explored risk factors for hyperuricemia [7–10]. A 6-year longitudinal study indicated that high blood pressure, high triglyceride level, obesity, and alcohol intake are contributory factors for the development of hyperuricemia in middle-aged Japanese men [8]. Our previous prospective study reported that triglyceride levels can independently predict the incidence of hyperuricemia [11]. Epidemiological records show that risk factors like hypertension, obesity, and high triglyceride often coexist and may significantly increase the risk of target organ damage [12]. However, most previous studies only examined the link between hyperuricemia and a single independent risk factor. We screened the risk factors (e.g., triglyceride, fasting plasma glucose, obesity, cholesterol, and blood pressure) for hyperuricemia in our cohort and found that hypertriglyceridemia (HTG) combined with hypertension (HTN) were more powerful in predicting the risk of hyperuricemia. Hence, hypertension and hypertriglyceridemia were selected to study.

In present study, we aimed to explore the separate and combined effects of hypertension and HTG on the incidence of hyperuricemia, and to fully adjust for potential confounders. We performed a cohort study in Tianjin, China, and conducted a routine physical examination each year from August 2009 to October 2017.

## Materials and methods

### Study population

This cohort study was performed at Tianjin General Hospital of China, which established in 2009 [13]. Individuals were invited to participate in annual physical examinations. Participants have undergone a range of physical measurements, detailed assessments about health-related factors, and sampling of blood. New participants were recruited to the dynamic cohort each year. Up to October 2017, 8962 participants were recruited, including 6799 males and 2163 females*.* Participants were excluded if they had hyperuricemia or had missing information on serum uric acid levels, blood pressure and TG levels. Ultimately, 6,424 subjects were included (Additional file 1: Fig. S1). All subjects provided written informed consent, and the protocol was approved by the Human Ethics Committee of Tianjin Medical University.

### Assessment and definitions of variates

Height and weight were measured according to a uniform standard and body mass index were calculated by Weight/ height squared (kg/m^2^). A well-trained nurse or doctor used a standard mercury sphygmomanometer to measure resting blood pressure in the right forearm for twice with the subjects rested for at least five minutes. Fasting serum uric acid, fasting plasma glucose, renal function (serum creatinine, blood urea nitrogen), liver function (total serum protein, aspartate aminotransferase, total bilirubin) and lipid profiles (triglyceride, total cholesterol) were obtained by laboratory examination.

Hypertension was defined as systolic blood pressure (SBP) ≥ 140 mmHg or diastolic blood pressure (DBP) ≥ 90 mmHg. Subjects current using of antihypertensive medication were automatically assumed into this category. HTG was defined as fasting serum TG levels > 150 mg/dl (1.7 mmol/L) [14]. According to the recommended criteria for Chinese people [15], a BMI of 18.5 to 23.9 was considered as optimal weight, 24.0 to 27.9 as overweight, and 28.0 and above as obese. Normal estimated glomerular filtration rate (eGFR) individuals with eGFR ≥ 60 ml/min/1.73  m^2^ [16]. Proteinuria, defined by urine dipstick result of 1 + or higher for protein.

### Outcome ascertainment

Hyperuricemia was defined as serum uric acid ≥ 420 μmol/L (7.0 mg/dl) in males and serum uric acid ≥ 360 μmol/L (6.0 mg/dl) in females [17]. Individuals who were found to have hyperuricemia without prior history were defined as incident cases.

### Statistical analysis

Continuous variables were described as mean and standard deviations (SD) and were determined with analysis of variance (ANOVA). Categorical variables were described as proportions and were determined with chi-squared tests. A series of Cox regression models were performed to calculate hazard ratios (HRs) and 95% confident intervals (CIs) to estimate baseline factors and their association with the incidence of hyperuricemia. The restricted cubic spline fitting Cox regression model was used to evaluate the relationship between different blood pressure and triglyceride cut-off value and hyperuricemia with four knots at the 25th, 50th, 75th, and 95th centiles. In the spline models, we adjusted age and taken 1.7 mmol/L, 130 mmHg and 80 mmHg as the reference value of TG, SBP and DBP, respectively.

All study subjects were divided into four groups by combining hypertension and HTG status at baseline: normotension and normal TG (normal group), hypertension and normal TG (separate hypertension), normotension and HTG (separate HTG), hypertension and HTG, respectively. We evaluated the risk of progression to hyperuricemia among the four groups, using Cox regression model and Kaplan–Meier survival analysis with adjustment for the potential confounders. Evaluation of blood pressure combined with triglyceride levels by Receiver operating characteristic curves (ROC) to obtain area under the curve (AUC) for predicting hyperuricemia. Subgroup analyses were conducted stratified by BMI (< 24, 24–27.9, ≥ 28 kg/m^2^) and eGFR (< 60, ≥ 60 ml/min/1.73  m^2^). All data were analyzed by SPSS version 17.0 (SPSS Inc., Chicago, IL, USA) and Stata version 13.0. Two-tailed *P* ≤ 0.05 indicated statistical significance.

## Results

In our cohort, 6,424 individuals were included, and the mean baseline age was 61.7 ± 13.7 years. Baseline characteristics of subjects by combining hyperuricemia and HTG status were shown in Table 1. Combination of hypertension and HTG tended to have higher serum uric acid levels, body mass index, serum creatinine, fasting plasma glucose, alanine aminotransferase and more proteinuria.

**Table 1:** Baseline characteristics of subjects by combining hypertension and hypertriglyceridemia status

Variables	Normotension and normal TG	Hypertension and normal TG	Normotension and HTG	Hypertension and HTG	*P*-value^*^
*N* (%)	2515 (39.2)	2028 (31.6)	907 (14.1)	974 (15.2)	
Age (years)	57.3 ± 13.5	68.4 ± 12.5	56.8 ± 11.6	64.1 ± 12.4	< 0.001
Men (%)	1704 (67.8)	1659 (81.8)	711 (78.4)	974 (79.8)	< 0.001
TG (mmol/L)	1.1 ± 0.3	1.1 ± 0.3	2.6 ± 1.3	2.6 ± 1.3	< 0.001
TC (mmol/L)	4.7 ± 0.8	4.8 ± 0.8	5.1 ± 0.9	5.1 ± 0.9	< 0.001
FPG (mmol/L)	5.1 ± 0.9	5.5 ± 1.3	5.3 ± 1.4	5.7 ± 1.7	< 0.001
SBP (mmHg)	120.9 ± 11.1	153.8 ± 19.9	123.0 ± 10.6	152.6 ± 14.4	< 0.001
DBP (mmHg)	69.8 ± 8.7	81.7 ± 11.8	72.5 ± 8.5	84.6 ± 12.0	< 0.001
SUA (µmol/L)	298.0 ± 60.9	308.6 ± 60.7	325.1 ± 56.1	332.5 ± 55.9	< 0.001
BUN (mmol/L)	5.1 ± 1.3	5.4 ± 1.3	5.1 ± 1.2	5.3 ± 1.2	< 0.001
SCR (µmol/L)	75.8 ± 15.7	81.3 ± 17.1	78.7 ± 14.1	81.4 ± 16.3	< 0.001
eGFR (ml/min/1.73 m^2^)	92.9 ± 18.3	86.4 ± 19.6	91.3 ± 16.6	86.8 ± 18.0	< 0.001
proteinuria (%)	62 (2.5)	80 (3.9)	33 (3.6)	53 (5.4)	< 0.001
TP (g/L)	73.4 ± 3.9	74.3 ± 4.3	74.7 ± 4.0	75.8 ± 4.1	< 0.001
ALT (IU/L)	21.2 ± 12.4	21.8 ± 12.8	26.4 ± 12.2	27.4 ± 16.0	< 0.001
TBIL (µmol/L)	13.7 ± 5.3	14.1 ± 5.0	13.3 ± 4.6	13.6 ± 4.4	0.057
BMI (kg/m^2^)	23.5 ± 2.9	24.8 ± 3.1	25.0 ± 2.7	26.1 ± 2.8	< 0.001

*TG* plasma triglyceride level, *TC* total cholesterol, *FPG* fasting plasma glucose, *SBP* systolic blood pressure, *DBP* diastolic blood pressure, *SUA* serum uric acid, *BUN* blood urea nitrogen, *SCR* serum creatinine, *eGFR* estimated glomerular filtration rate, *TP* plasma total protein, *ALT* alanine aminotransferase, *TBIL* total bilirubin, *BMI* body mass index, *HTG* hypertriglyceridemia

^*^P < 0.05 was considered statistically significant

Over a period of 8 years, 1259 individuals diagnosed with hyperuricemia and the cumulative incidence of hyperuricemia was 20.2%, 21.3% for men and 16.6% for women. Furthermore, regardless of men or women, the incidence of hyperuricemia among individuals with a combination of hypertension and HTG (37.8%, 95% CI 34.8–40.9%) was significantly higher than that of those with hypertension alone (18.7%, 95% CI 17.0–20.4%), HTG alone (24.5%, 95% CI 21.7–27.3%) and neither hypertension nor HTG group (13.0%, 95% CI 11.7%-14.3%) (Additional file 1: Fig. S2).

Our results revealed that there existed positive relationships between blood pressure and triglyceride level and the incidence of hyperuricemia assessed by the restricted cubic spline fitting Cox model (Additional file 1: Fig. S3). After adjusted age, with the increase of blood pressure, the risk of hyperuricemia increases gradually (Additional file 1: Fig. S3a, b, d). Our results also suggested that with the increase of triglyceride levels, the risk of hyperuricemia increases gradually and then stabilizes (Additional file 1: Fig. S3 g, h, i), and triglyceride level at around 2.5 mmol/l cut-off point has the greatest hyperuricemia risk.

Cox regression model assessed the impact of comorbid hypertension and HTG on the risk of hyperuricemia (Table 2). In an unadjusted model of overall participant, the HRs (95% *CI*s) for hyperuricemia in the combination of hypertension and HTG, HTG alone and hypertension alone groups, compared with the normal control group (normotension and normal TG), were 1.48 (1.28–1.71), 1.84 (1.55–2.18), and 3.02 (2.60–3.50), respectively; after adjusting for sex and age, the HRs (95% *CI*s) were 1.39 (1.19–1.63), 1.80 (1.52–2.14), and 2.88 (2.48–3.36), respectively. After further adjustment for other confounders, the association also found to be significant, with HRs (95% *CI*s) of 1.24 (1.05–1.46), 1.61 (1.34–1.93), and 2.36 (2.00–2.78), respectively. Additionally, after fully adjustment for potential confounders, the HRs for hyperuricemia in the combination of hypertension and HTG, HTG alone and hypertension alone groups, compared with the normal control group, for men, were 1.17 (0.98–1.40), 1.43 (1.17–1.76), 2.22 (1.85–2.67), respectively; and for women, were 1.57 (1.07–2.32), 2.36 (1.60–3.47), 2.76 (1.87–4.07), respectively.

**Table 2:** Hazard ratio for the incidence of hyperuricemia by combining hypertension and hypertriglyceridemia status

	Hazard Ratio (95% *CI*)				*P*-value for trend
	Normotension and normal TG	Hypertension and normal TG	Normotension and HTG	Hypertension and HTG	
Overall^a^					
Model 1	1 (Ref.)	1.48 (1.28–1.71)	1.84 (1.55–2.18)	3.02 (2.60–3.50)	< 0.001
Model 2	1 (Ref.)	1.39 (1.19–1.63)	1.80 (1.52–2.14)	2.88 (2.48–3.36)	< 0.001
Model 3	1 (Ref.)	1.24 (1.05–1.46)	1.61 (1.34–1.93)	2.36 (2.00–2.78)	< 0.001
Men^b^					
Model 1	1 (Ref.)	1.28 (1.09–1.52)	1.58 (1.30–1.91)	2.63 (2.23–3.11)	< 0.001
Model 2	1 (Ref.)	1.28 (1.08–1.51)	1.57 (1.29–1.91)	2.63 (2.22–3.11)	< 0.001
Model 3	1 (Ref.)	1.17 (0.98–1.40)	1.43 (1.17–1.76)	2.22 (1.85–2.67)	< 0.001
Women^b^					
Model 1	1 (Ref.)	2.06 (1.48–2.87)	2.77 (1.93–3.98)	4.16 (3.00–5.76)	< 0.001
Model 2	1 (Ref.)	1.77 (1.23–2.56)	2.62 (1.82–3.78)	3.60 (2.52–5.16)	< 0.001
Model 3	1 (Ref.)	1.57 (1.07–2.32)	2.36 (1.60–3.47)	2.76 (1.87–4.07)	< 0.001

^a^Model 1 was unadjusted baseline values of variables. Model 2 was adjustment for age and sex. Model 3 was further adjustment for body-mass index, eGFR, blood urea nitrogen and proteinuria

^b^Model 1 was unadjusted baseline values of variables. Model 2 was adjustment for age. Model 3 was further adjustment for body-mass index, eGFR, blood urea nitrogen and proteinuria

**Fig. 1:**
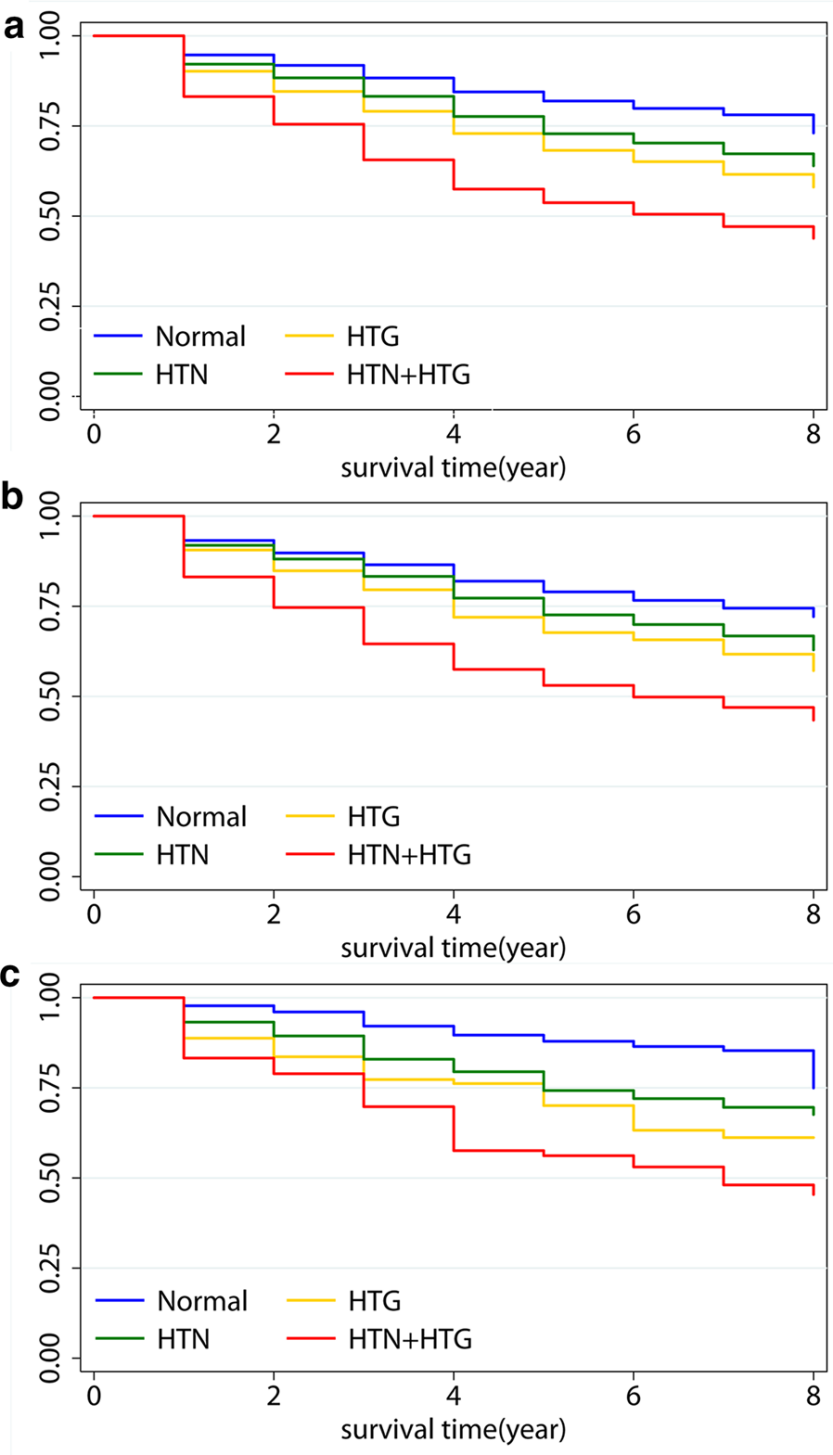
Kaplan‑Meier curves for the incidence of hyperuricemia among four groups in overall (**a**); in men (**b**), and in women (**c**). (log‑rank test *P*-value < 0.05, HTN + HTG group vs. other groups). Normal, normotension and normal triglyceride; HTN, hypertension and normal triglyceride; HTG: normotension and hypertriglyceridemia; HTN + HTG, hypertension and hypertriglyceridemia

Survival analysis of the risk of hyperuricemia for men and women in four groups during the 8-year period were showed in Fig. 1. Kaplan–Meier survival curve showed that hypertension combined with HTG could predict the highest risk of hyperuricemia in four groups of men and women.

To explore effect of obesity and impaired renal function on hypertension and HTG correlations, this cohort stratified by BMI (< 24, 24–27.9, ≥ 28 kg/m^2^) and eGFR (< 60, ≥ 60 ml/min/1.73 m^2^). The Cox regression results with hyperuricemia risk were relatively unchanged in individuals of normal-weight and normal-eGFR (Table 3).

**Table 3:** Stratified Analysis of multivariate hazard ratios for hyperuricemia among 6424 individuals according to combining hypertension and hypertriglyceridemia status

	Hazard Ratio (95% *CI*)				*P*-Value for trend	*P-*Value for Interaction
	Normotension and normal TG	Hypertension and normal TG	Normotension and HTG	Hypertension and HTG		
Body-mass index						
< 24	1	1.46 (1.12–1.92)	1.96 (1.45–2.64)	2.49 (1.81–3.44)	< 0.001	0.80
24 ~ 27.9	1	1.16 (0.92–1.47)	1.51 (1.17–1.95)	2.24 (1.79–2.82)	< 0.001	
≥ 28	1	1.06 (0.68–1.66)	1.30 (0.78–2.17)	2.40 (1.58–3.66)	< 0.001	
eGFR (ml/min/1.73 m^2^)						0.60
< 60	1	1.17 (0.37–3.78)	1.70 (0.75–3.86)	3.63 (1.58–5.33)	< 0.001	
≥ 60	1	1.24 (1.04–1.48)	1.65 (1.37–2.00)	2.34 (1.96–2.78)	< 0.001	

The Cox regression analysis was adjusted, if not stratified, for age, body-mass index, and blood urea nitrogen, as continuous variables; eGFR (< 60 or ≥ 60 ml/min/1.73 m^2^ or missing information); and proteinuria (positive, negative, or missing information)

**Fig. 2:**
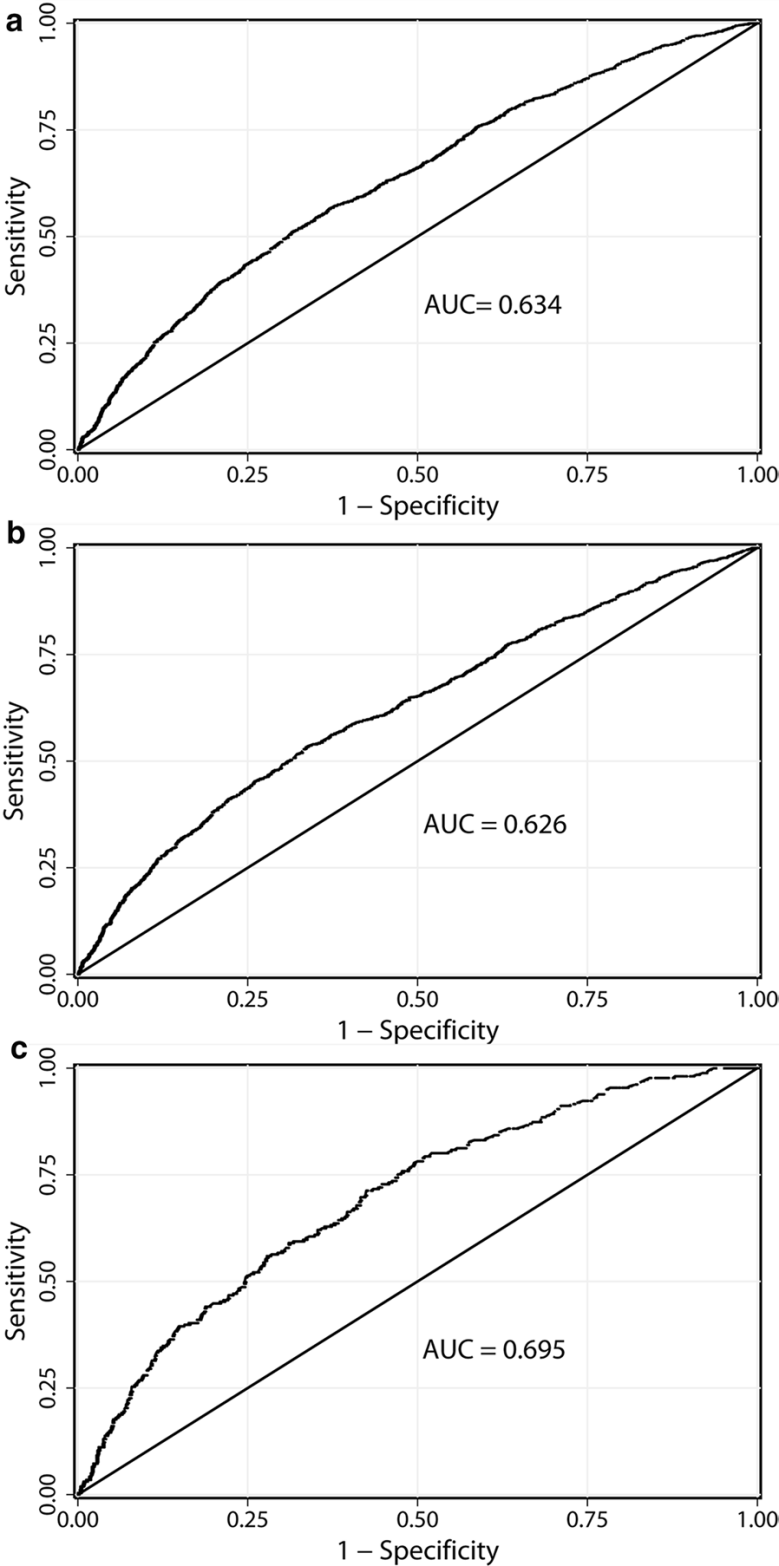
Receiver operating characteristic (ROC) curve for the prediction of hyperuricemia from blood pressure combined with TG levels in overall (**a**); in men (**b**), and in women (**c**). AUC, area under the curve. All models AUC > 0.500, *P* < 0.001

ROC analysis showed an AUC of 0.634 in the total cohort, 57.6% for sensitivity and 63.4% for specificity. For men, the AUC was 0.626, 59.1% for sensitivity and 61.7% for specificity. For women, the AUC was 0.695, 71.3% for sensitivity and 57.5% for specificity (Fig. 2a–c). All the above models were significantly different from the random predictor (AUC > 0.500, *P* < 0.001).

## Discussion

Our findings suggest that hypertension combined with HTG can significantly increase the incidence of hyperuricemia more than the presence of hypertension or HTG alone can. These combined effects of hypertension and HTG on the incidence of hyperuricemia remained significant even after adjusting for potential confounders. Moreover, there existed positive relationships between blood pressure and triglyceride level and the incidence of hyperuricemia assessed by the restricted cubic spline fitting Cox model. Additionally, the combined effect of hypertension and HTG were more strongly associated with hyperuricemia among women than among men.

Our findings relating hypertension and HTG to the risk of developing hyperuricemia support some previous studies. Our results are similar to observations in cross-sectional analyses and prospective cohort studies [9, 18–20]. A cross-sectional survey, including 11,576 Chinese rural adults found that both hypertension and HTG were significantly associated with hyperuricemia risk [18]. The ARIC cohort study found that after fully adjusting for confounding factors, hypertension and high TG levels could predict the incidence of hyperuricemia, with hazard ratios of 1.65 and 2.00, respectively [9]. Our reported hazard ratios (hypertension: 1.24, and TG:1.61) were lower than those in the ARIC cohort study, and the cause of this difference might be our older study population. Moreover, a cohort study of hyperuricemia reported that the incidence of hyperuricemia increased across baseline categories of hypertension (hazard ratio, 1.19) and high TG levels (hazard ratio, 1.47) [21], which were comparable to ours. Nevertheless, epidemiological records show that hypertension and high triglyceride often coexist and may significantly increase the risk of target organ damage. However, as previously stated, most prior studies have concentrated solely on examining the link between hypertension or HTG and the risk of hyperuricemia on an individual basis.

Both hypertension and HTG are important components of metabolic syndrome. Studies have reported the association of metabolic syndrome and hyperuricemia [22, 23]. However, there are few studies to explore the combined effect of hypertension and HTG on incident hyperuricemia. We found that hypertension combined with HTG could significantly increase the risk of developing hyperuricemia than any separate hypertension or HTG. Insulin resistance plays a potentially key role in the relationship between hypertension and HTG and the risk of developing hyperuricemia. Previous studies showed that insulin resistance is partially overlapping pathophysiological features of dyslipidemia and hypertension [24–27]. Much researches have confirmed that insulin resistance was correlated with hyperuricemia [28, 29]. Battelli et al. [30] reported that hyperinsulinemia increases urate reabsorption in the proximal tubule and causes hyperuricemia. A prospective study in 2071 non-diabetic Japanese men found that insulin resistance itself or compensatory hyperinsulinemia may contribute to the development of hyperuricemia [28]. Therefore, insulin resistance may be the common pathway for the development of hyperuricemia in instances where the subject shows a combination of hypertension and HTG. In addition, hypertension and HTG are manifestations of the metabolic syndrome and they frequently coexist [12]. Some prospective studies suggest that hypertension may be a consequence of dyslipidemia [31]. Laaksonen et al. [32] also reported that a 1-SD increment in triglyceride levels was correlated with a 1.6-fold increased risk of developing hypertension, independent of features related to the metabolic syndrome. There may be some complicated effects between the pathogenesis of hypertension and triglycerides, which is worth further exploration.

The present study also suggests that the combined effect of hypertension and HTG were more strongly associated with hyperuricemia among women than among men. The potential mechanism may be serum uric acid levels in women increase with aging after menopause because female hormones decrease with aging [33]. Our study had an older population (mean age of 61.7 years). After menopause, due to declining estrogen, the risk for some cardiovascular diseases increase [34]. Similarly, loss of the protection from estrogen, the combined effect of hypertension and HTG may have a higher risk for hyperuricemia in women than in men.

Hypertension and HTG are driven by many factors, and we suspect that these contribute to the relationship between hypertension combined with HTG and the risk of hyperuricemia. Some studies reported that BMI and renal function were associated with both hypertension and TG levels [35–37]. However, our stratified analysis of these factors suggested that the association was not modified by any of these variables, suggesting an independent association between hypertension combined with HTG and the incidence of hyperuricemia.

The strengths of our research include a dynamic cohort study, a long follow-up period and a large population sample. Furthermore, this study involved detailed demographic and clinical information, allowing to adjust for potential confounders. Additionally, our study focuses on the elderly, who happen to be at high risk of chronic diseases, which is conducive to our research. As far as is known, this is the first cohort study to assess the combined effect of hypertension and HTG on the incidence of hyperuricemia. This study also has several limitations. First, we lack data on alcohol consumption, dietary habits, lifestyle factors that were known to have an impact on incident hyperuricemia. Second, we did not have detailed information on medications, such as diuretic, which may affect our results. Third, most of our participants were university teachers and employees of government-funded institutions, which were more likely to have a higher socioeconomic status. Therefore, our participants may not be fully representative of the general population. Thus, it is necessary to further verify our findings in the general population.

## Conclusion

Our findings suggest that the combination of hypertension and HTG has greater effect on hyperuricemia than any separate hypertension or HTG. Moreover, hypertension combined with HTG can independently and powerfully predict the incidence of hyperuricemia. These data can provide important information for identifying individuals who are at high risk of hyperuricemia, which helps early prevention of the disease and subsequent gouty arthritis.

## Supplementary information

**Additional file 1: Figure S1.** Flow chart of participants included in the study. **Table S1.** Baseline characteristics and incident hyperuricemia by univariate Cox regression. **Figure S2.** Cumulative incidence of hyperuricemia among men and women by combining hypertension and hypertriglyceridemia status. Normal, normotension and normal triglyceride; HTN, hypertension and normal triglyceride; HTG: normotension and hypertriglyceridemia; HTN+HTG, hypertension and hypertriglyceridemia. **Figure S3.** Age-adjusted relationship of systolic blood pressure (SBP), diastolic blood pressure (DBP) and plasma triglyceride (TG) levels with risk of hyperuricemia, evaluated using restricted cubic splines. (a) SBP and hyperuricemia among overall; (b) SBP and hyperuricemia among men; (c) SBP and hyperuricemia among women; (d) DBP and hyperuricemia among overall; (e) DBP and hyperuricemia among men; (f) DBP and hyperuricemia among women; (g) TG and hyperuricemia among overall; (h) TG and hyperuricemia among men; (i) SBP and hyperuricemia among women; Hazard ratios are indicated by solid lines and 95% CIs by shaded areas. Reference point is 1.7 mmol/L for TG, 130mmHg for SBP and 80mmHg for DBP, with four knots at the 25th, 50th, 75th, and 95th centiles.

## Data Availability

All the data and materials used in our article are available from the corresponding author on reasonable request.

## References

[1] Liu R, Han C, Wu D, Xia X, Gu J, Guan H, Shan Z, Teng W (2015). Prevalence of hyperuricemia and gout in Mainland China from 2000 to 2014: a systematic review and meta-analysis. Biomed Res Int.

[2] Zhu Y, Pandya BJ, Choi HK (2011). Prevalence of gout and hyperuricemia in the US general population: the National Health and Nutrition Examination Survey 2007–2008. Arthritis Rheum.

[3] Nagahama K, Iseki K, Inoue T, Touma T, Ikemiya Y, Takishita S (2004). Hyperuricemia and cardiovascular risk factor clustering in a screened cohort in Okinawa Japan. Hypertens Res.

[4] Krishnan E, Pandya BJ, Chung L, Dabbous O (2011). Hyperuricemia and the risk for subclinical coronary atherosclerosis–data from a prospective observational cohort study. Arthritis Res Ther.

[5] Krishnan E, Pandya BJ, Lingala B, Hariri A, Dabbous O (2012). Hyperuricemia and untreated gout are poor prognostic markers among those with a recent acute myocardial infarction. Arthritis Res Ther.

[6] Baker JF, Krishnan E, Chen L, Schumacher HR (2005). Serum uric acid and cardiovascular disease: recent developments, and where do they leave us?. Am J Med.

[7] Liu H, Zhang XM, Wang YL, Liu BC (2014). Prevalence of hyperuricemia among Chinese adults: a national cross-sectional survey using multistage, stratified sampling. J Nephrol.

[8] Nakanishi N, Tatara K, Nakamura K, Suzuki K (1999). Risk factors for the incidence of hyperuricaemia: a 6-year longitudinal study of middle-aged Japanese men. Int J Epidemiol.

[9] McAdams-DeMarco MA, Law A, Maynard JW, Coresh J, Baer AN (2013). Risk factors for incident hyperuricemia during mid-adulthood in African American and white men and women enrolled in the ARIC cohort study. BMC Musculoskelet Disord.

[10] Wu J, Qiu L, Cheng XQ, Xu T, Wu W, Zeng XJ, Ye YC, Guo XZ, Cheng Q, Liu Q (2017). Hyperuricemia and clustering of cardiovascular risk factors in the Chinese adult population. Sci Rep.

[11] Zhang Y, Wei F, Chen C, Cai C, Zhang K, Sun N, Tian J, Shi W, Zhang M, Zang Y (2018). Higher triglyceride level predicts hyperuricemia: a prospective study of 6-year follow-up. J Clin Lipidol.

[12] Eckel RH, Alberti KG, Grundy SM, Zimmet PZ (2010). The metabolic syndrome. Lancet.

[13] Zhao J, Zhang Y, Wei F, Song J, Cao Z, Chen C, Zhang K, Feng S, Wang Y, Li WD (2019). Triglyceride is an independent predictor of type 2 diabetes among middle-aged and older adults: a prospective study with 8-year follow-ups in two cohorts. J Transl Med.

[14] Tenenbaum A, Klempfner R, Fisman EZ (2014). Hypertriglyceridemia: a too long unfairly neglected major cardiovascular risk factor. Cardiovasc Diabetol.

[15] Wu Y (2006). Overweight and obesity in China. BMJ.

[16] Van Pottelbergh G, Vaes B, Adriaensen W, Mathei C, Legrand D, Wallemacq P, Degryse JM (2014). The glomerular filtration rate estimated by new and old equations as a predictor of important outcomes in elderly patients. BMC Med.

[17] Wei F, Sun N, Cai C, Feng S, Tian J, Shi W, Xu W, Wang Y, Yang X, Li WD (2016). Associations between serum uric acid and the incidence of hypertension: a Chinese senior dynamic cohort study. J Transl Med.

[18] Yu S, Yang H, Guo X, Zhang X, Zhou Y, Ou Q, Zheng L, Sun Y (2016). Prevalence of hyperuricemia and its correlates in rural Northeast Chinese population: from lifestyle risk factors to metabolic comorbidities. Clin Rheumatol.

[19] Peng TC, Wang CC, Kao TW, Chan JY, Yang YH, Chang YW, Chen WL (2015). Relationship between hyperuricemia and lipid profiles in US adults. Biomed Res Int.

[20] Li X, Song P, Li J, Wang P, Li G (2015). Relationship between hyperuricemia and dietary risk factors in Chinese adults: a cross-sectional study. Rheumatol Int.

[21] Ryu S, Chang Y, Zhang Y, Kim SG, Cho J, Son HJ, Shin H, Guallar E (2012). A cohort study of hyperuricemia in middle-aged South Korean men. Am J Epidemiol.

[22] Li C, Hsieh MC, Chang SJ (2013). Metabolic syndrome, diabetes, and hyperuricemia. Curr Opin Rheumatol.

[23] Wang H, Zhang H, Sun L, Guo W (2018). Roles of hyperuricemia in metabolic syndrome and cardiac-kidney-vascular system diseases. Am J Transl Res.

[24] Esteghamati A, Khalilzadeh O, Abbasi M, Nakhjavani M, Novin L, Esteghamati AR (2008). HOMA-estimated insulin resistance is associated with hypertension in Iranian diabetic and non-diabetic subjects. Clin Exp Hypertens.

[25] Akande TO, Adeleye JO, Kadiri S (2013). Insulin resistance in Nigerians with essential hypertension. Afr Health Sci.

[26] Shulman GI (2014). Ectopic fat in insulin resistance, dyslipidemia, and cardiometabolic disease. N Engl J Med.

[27] Skarn SN, Flaa A, Kjeldsen SE, Rostrup M, Brunborg C, Reims HM, Fossum E, Hoieggen A, Aksnes TA (2015). Family history of hypertension and serum triglycerides predict future insulin sensitivity: a 17-year follow-up study of young men. J Hypertens.

[28] Nakamura K, Sakurai M, Miura K, Morikawa Y, Nagasawa SY, Ishizaki M, Kido T, Naruse Y, Nakashima M, Nogawa K (2014). HOMA-IR and the risk of hyperuricemia: a prospective study in non-diabetic Japanese men. Diabetes Res Clin Pract.

[29] Chen LK, Lin MH, Lai HY, Hwang SJ, Chiou ST (2008). Uric acid: a surrogate of insulin resistance in older women. Maturitas.

[30] Battelli MG, Bortolotti M, Polito L, Bolognesi A (2018). The role of xanthine oxidoreductase and uric acid in metabolic syndrome. Biochim Biophys Acta Mol Basis Dis.

[31] Sesso HD, Buring JE, Chown MJ, Ridker PM, Gaziano JM (2005). A prospective study of plasma lipid levels and hypertension in women. Arch Intern Med.

[32] Laaksonen DE, Niskanen L, Nyyssonen K, Lakka TA, Laukkanen JA, Salonen JT (2008). Dyslipidaemia as a predictor of hypertension in middle-aged men. Eur Heart J.

[33] Liu B, Wang T, Zhao HN, Yue WW, Yu HP, Liu CX, Yin J, Jia RY, Nie HW (2011). The prevalence of hyperuricemia in China: a meta-analysis. BMC Public Health.

[34] Hak AE, Choi HK (2008). Menopause, postmenopausal hormone use and serum uric acid levels in US women—the Third National Health and Nutrition Examination Survey. Arthritis Res Ther.

[35] Jackson C, Herber-Gast GC, Brown W (2014). Joint effects of physical activity and BMI on risk of hypertension in women: a longitudinal study. J Obes.

[36] Banach M, Aronow WS, Serban MC, Rysz J, Voroneanu L, Covic A (2015). Lipids, blood pressure and kidney update 2015. Lipids Health Dis.

[37] Zubovic SV, Kristic S, Prevljak S, Pasic IS (2016). Chronic kidney disease and lipid disorders. Med Arch.

