# Supplementary material for: Association of hypertension and hypertriglyceridemia on incident hyperuricemia: an 8-year prospective cohort study
Source: J Transl Med. 2020 Oct 31;18:409. doi: 10.1186/s12967-020-02590-8 (PMC7603698; doi:10.1186/s12967-020-02590-8)

**Additional file**

**Zhang et al. Association of hypertension and hypertriglyceridemia on incident hyperuricemia: An 8-year prospective cohort study**

**Figure S1.** Flow chart of participants included in the study


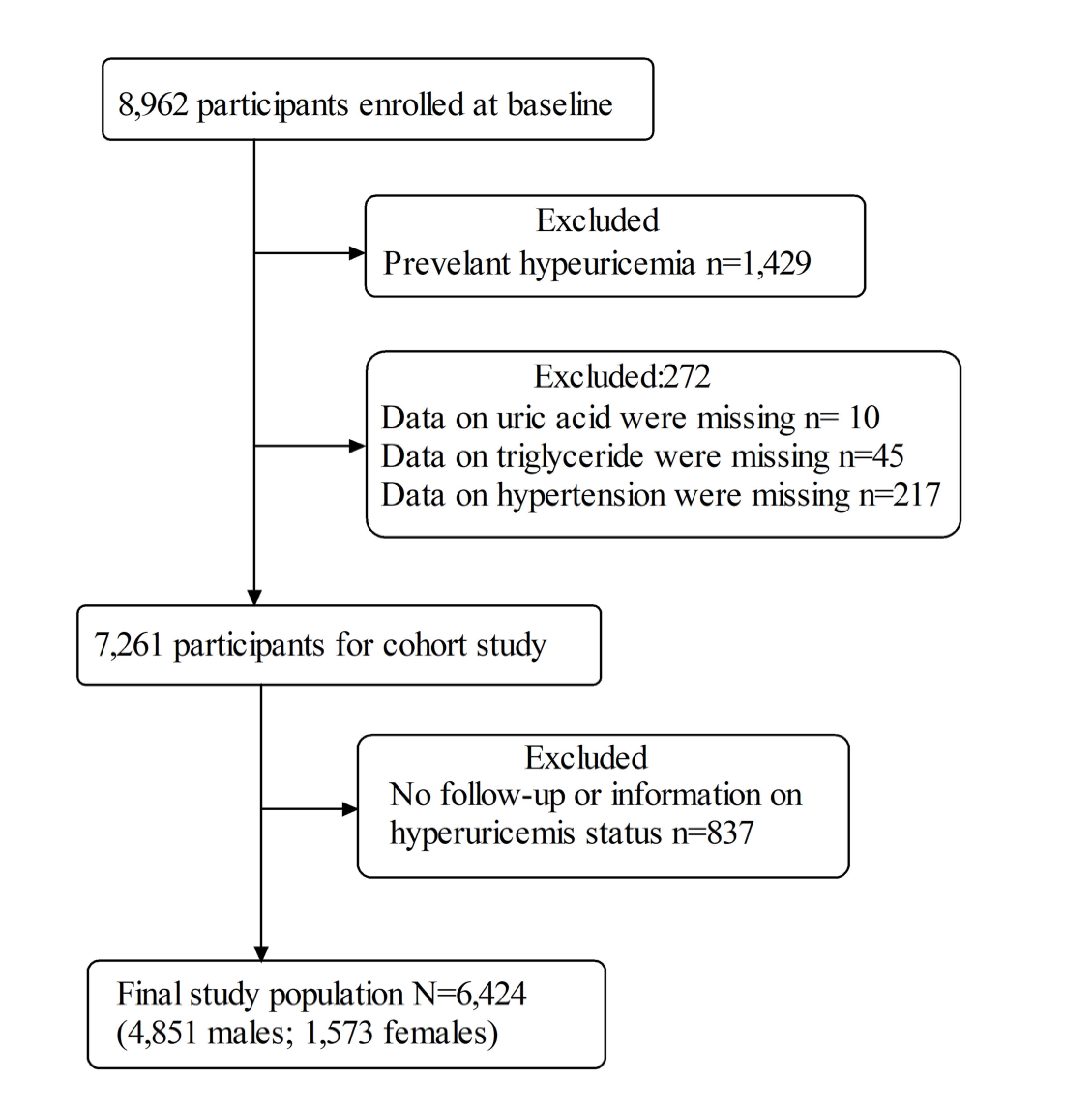


**Table S1.** Baseline characteristics and incident hyperuricemia by univariate Cox regression

| Variables | HR (95% CI) | *P* value |
| --- | --- | --- |
| Age (years) | 1.01 (1.00-1.02) | 0.002 |
| Men (%) | 1.35 (1.18-1.55) | <0.001 |
| TG (mmol/L) | 1.19 (1.16-1.22) | <0.001 |
| TC (mmol/L) | 1.03 (0.96-1.11) | 0.362 |
| FPG (mmol/L) | 1.01 (0.97-1.05) | 0.711 |
| SBP (mmHg) | 1.01 (1.01-1.02) | <0.001 |
| DBP (mmHg) | 1.02 (1.01-1.02) | <0.001 |
| SUA (µmol/L) | 1.02 (1.01-1.02) | <0.001 |
| BUN(mmol/L) | 1.09 (1.04-1.13) | <0.001 |
| eGFR (ml/min/1.73 m2) | 0.98 (0.98-0.99) | <0.001 |
| proteinuria (%) | 1.40 (1.08-1.81) | 0.010 |
| TP (g/L) | 1.04 (0.98-1.06) | 0.214 |
| ALT (IU/L) | 1.00 (0.99-1.00) | 0.083 |
| BMI (kg/m2) | 1.12 (1.10-1.14) | <0.001 |

Abbreviations: TG, plasma triglyceride level; TC, total cholesterol; FPG, fasting plasma glucose; SBP, systolic blood pressure; DBP, diastolic blood pressure; BUN, blood urea nitrogen; SCR, serum creatinine; eGFR, estimated glomerular filtration rate; TP, plasma total protein; ALT, alanine aminotransferase; BMI, body mass index.

^*^*P*< 0.05 was considered statistically significant.

**Figure S2.** Cumulative incidence of hyperuricemia among men and women by combining hypertension and hypertriglyceridemia status. Normal, normotension and normal triglyceride; HTN, hypertension and normal triglyceride; HTG: normotension and hypertriglyceridemia; HTN+HTG, hypertension and hypertriglyceridemia.


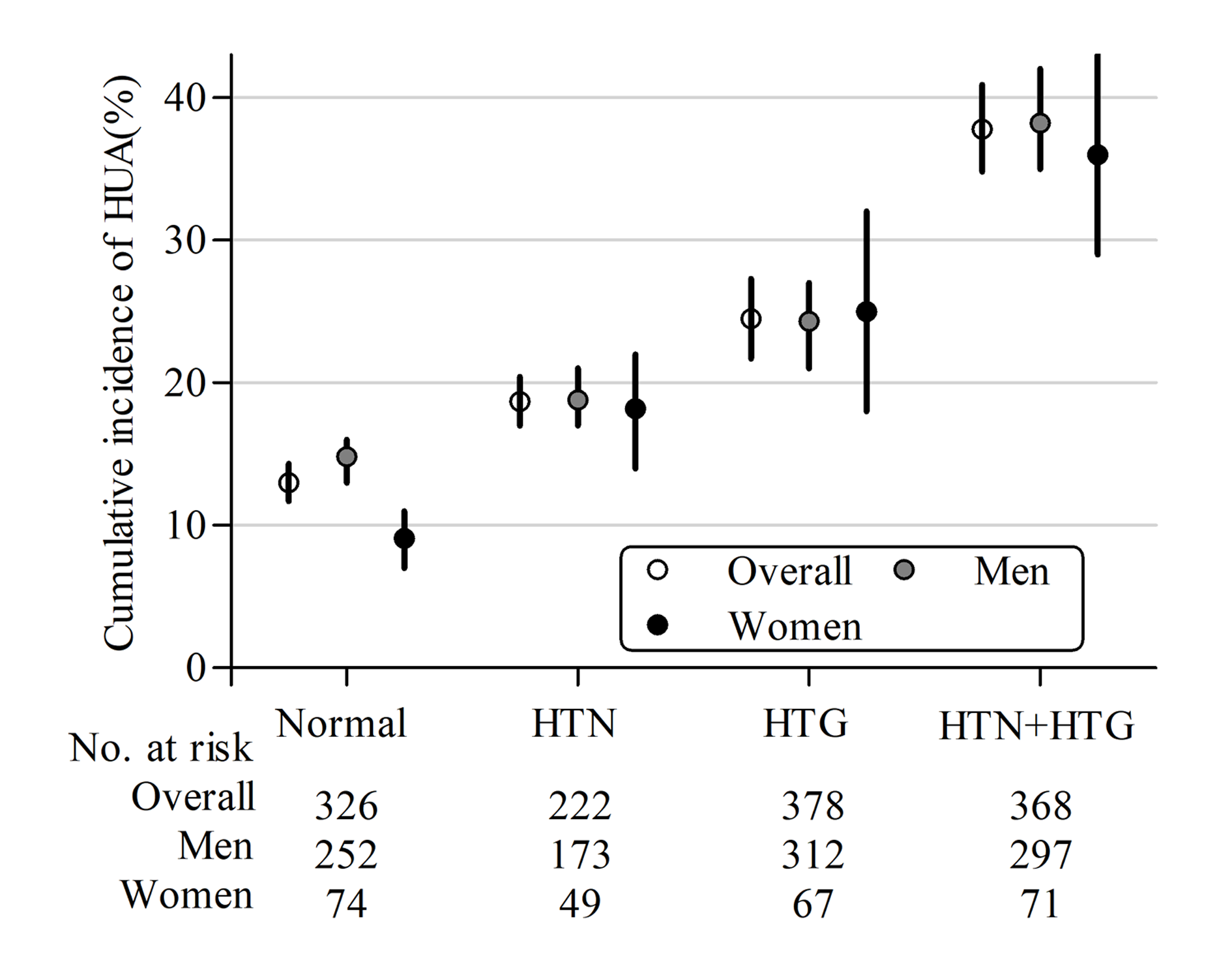


**Figure S3.** Age-adjusted relationship of systolic blood pressure (SBP), diastolic blood pressure (DBP) and plasma triglyceride (TG) levels with risk of hyperuricemia, evaluated using restricted cubic splines. (**a**) SBP and hyperuricemia among overall; (**b**) SBP and hyperuricemia among men; (**c**) SBP and hyperuricemia among women; (**d)** DBP and hyperuricemia among overall; (**e**) DBP and hyperuricemia among men; (**f**) DBP and hyperuricemia among women; (**g)** TG and hyperuricemia among overall; (**h**) TG and hyperuricemia among men; (**i**) SBP and hyperuricemia among women; Hazard ratios are indicated by solid lines and 95% CIs by shaded areas. Reference point is 1.7mmol/L for TG, 130mmHg for SBP and 80mmHg for DBP, with four knots at the 25th, 50th, 75th, and 95th centiles.


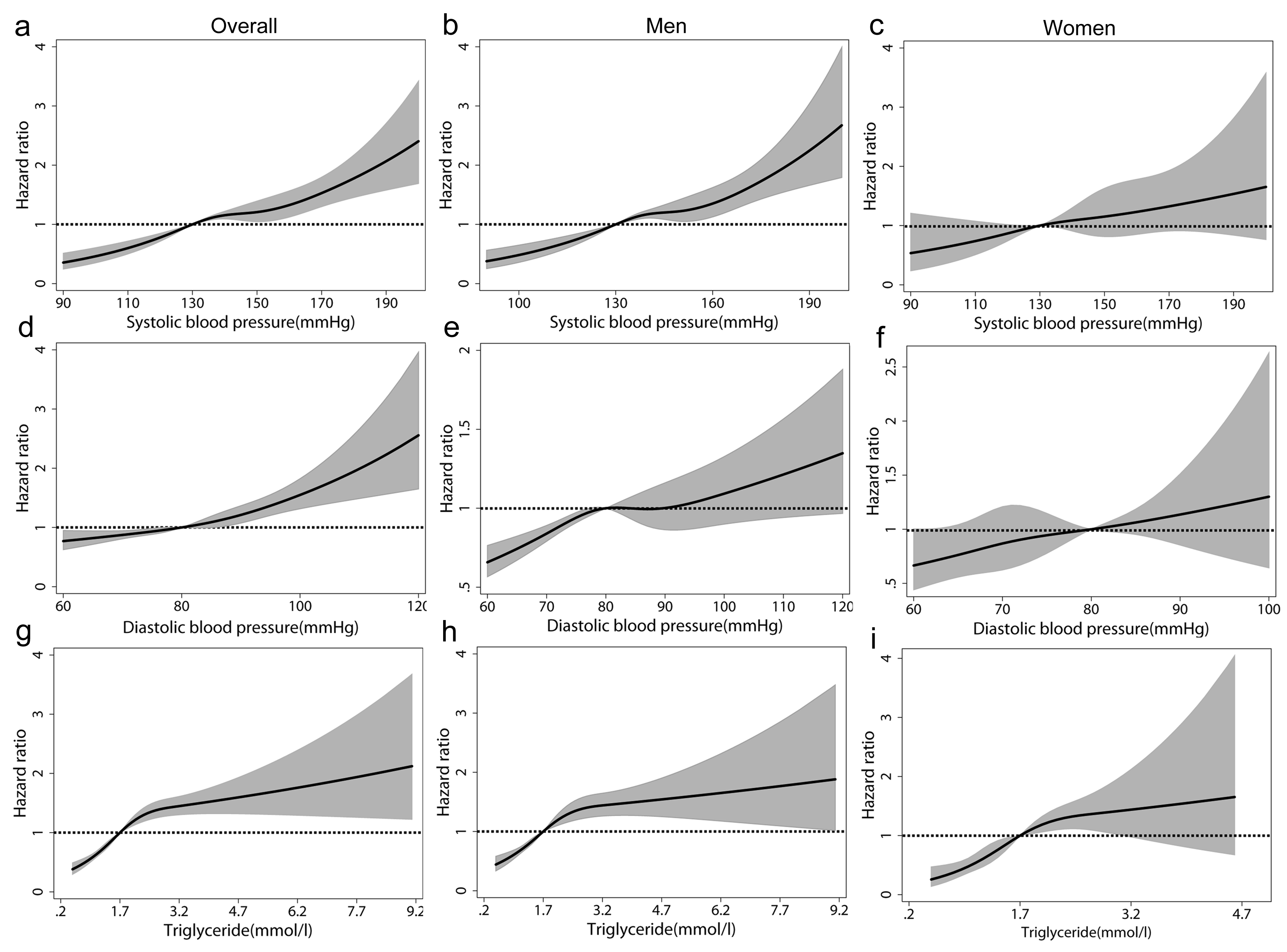

Supplement: Supplementary file 1 — Additional file 1: Figure S1. Flow chart of participants included in the study. Table S1. Baseline characteristics and incident hyperuricemia by univariate Cox regression. Figure S2. Cumulative incidence of hyperuricemia among men and women by combining hypertension and hypertriglyceridemia status. Normal, normotension and normal triglyceride; HTN, hypertension and normal triglyceride; HTG: normotension and hypertriglyceridemia; HTN+HTG, hypertension and hypertriglyceridemia. Figure S3. Age-adjusted relationship of systolic blood pressure (SBP), diastolic blood pressure (DBP) and plasma triglyceride (TG) levels with risk of hyperuricemia, evaluated using restricted cubic splines. (a) SBP and hyperuricemia among overall; (b) SBP and hyperuricemia among men; (c) SBP and hyperuricemia among women; (d) DBP and hyperuricemia among overall; (e) DBP and hyperuricemia among men; (f) DBP and hyperuricemia among women; (g) TG and hyperuricemia among overall; (h) TG and hyperuricemia among men; (i) SBP and hyperuricemia among women; Hazard ratios are indicated by solid lines and 95% CIs by shaded areas. Reference point is 1.7 mmol/L for TG, 130mmHg for SBP and 80mmHg for DBP, with four knots at the 25th, 50th, 75th, and 95th centiles. [file 12967_2020_2590_MOESM1_ESM.doc]
